# Novel Gene Fusions in Glioblastoma Tumor Tissue and Matched Patient Plasma

**DOI:** 10.3390/cancers12051219

**Published:** 2020-05-13

**Authors:** Lan Wang, Anudeep Yekula, Koushik Muralidharan, Julia L. Small, Zachary S. Rosh, Keiko M. Kang, Bob S. Carter, Leonora Balaj

**Affiliations:** 1 Department of Neurosurgery, Massachusetts General Hospital and Harvard Medical School, Boston, MA 02115, USA; lanwang.amber@gmail.com (L.W.); ayekula@mgh.harvard.edu (A.Y.); kmuralidharan@mgh.harvard.edu (K.M.); jsmall6@mgh.harvard.edu (J.L.S.); Zrosh@mgh.harvard.edu (Z.S.R.); kmkang@mgh.harvard.edu (K.M.K.); 2 School of Medicine, University of California San Diego, San Diego, CA 92092, USA

**Keywords:** glioblastoma, gene fusions, liquid biopsy, RNA-seq, extracellular vesicles

## Abstract

Sequencing studies have provided novel insights into the heterogeneous molecular landscape of glioblastoma (GBM), unveiling a subset of patients with gene fusions. Tissue biopsy is highly invasive, limited by sampling frequency and incompletely representative of intra-tumor heterogeneity. Extracellular vesicle-based liquid biopsy provides a minimally invasive alternative to diagnose and monitor tumor-specific molecular aberrations in patient biofluids. Here, we used targeted RNA sequencing to screen GBM tissue and the matched plasma of patients (*n* = 9) for RNA fusion transcripts. We identified two novel fusion transcripts in GBM tissue and five novel fusions in the matched plasma of GBM patients. The fusion transcripts FGFR3-TACC3 and VTI1A-TCF7L2 were detected in both tissue and matched plasma. A longitudinal follow-up of a GBM patient with a FGFR3-TACC3 positive glioma revealed the potential of monitoring RNA fusions in plasma. In summary, we report a sensitive RNA-seq-based liquid biopsy strategy to detect RNA level fusion status in the plasma of GBM patients.

## 1. Introduction

Glioblastoma Multiforme (GBM) is the most common malignant primary central nervous system tumor. It is highly aggressive, with a median overall survival of 15–23 months despite aggressive treatment [1]. A recent large scale genomic and transcriptomic sequencing analysis has shed light on the heterogeneous molecular landscape of GBM, with approximately 30–50% of patients harboring gene fusions [2]. Gene fusions are chromosomal alterations formed as a result of translocation, interstitial deletions, or insertions, resulting in a hybrid of two coding or regulatory sequences between genes [3]. Fusion transcripts are hybrid RNA produced as a result of chromosomal rearrangement (DNA level) or trans-splicing and cis splicing between adjacent genes (RNA level), and have been associated with cancer. These, in turn, generate chimeric proteins with altered functions to drive oncogenic pathways [4].

Emerging studies in cancers have demonstrated the role of oncogenic fusions, such as EML4-ALK in non-small-cell lung cancer [5], BCR-ABL1 fusion in chronic myeloid leukemia [6,7], PML-RARA in acute promyelocytic leukemia [8] and FGFR3-TACC3 in GBM [9] in tumor growth and progression. These fusions have been extensively described in literature from a diagnostic, prognostic and therapeutic standpoint [10]. Therefore, identifying and understanding gene fusions in glioma patients is critical in developing personalized diagnostic, monitoring and therapeutic modalities.

Conventionally, tumor tissue biopsy is used for histological and molecular analysis, including fusion identification [11]. In the context of CNS tumors, this procedure is highly invasive and serial biopsies are not practical to capture temporal and spatial tumor heterogeneity [12,13,14]. Liquid biopsy offers a minimally invasive platform for the molecular profiling of cancers from biofluids as a potential means to diagnose and monitor cancers over time and therapy [12,13,14,15]. Extracellular vesicles (EVs) are small membrane-bound nanoparticles released by all cells, including cancer cells, and have emerged as an attractive liquid biopsy modality due to their stable architecture that encloses tumor-specific genetic cargo, including mRNA, miRNA and other non-coding RNA which are reflective of the cell of origin [16]. Myriad studies over the past decade have demonstrated the utility of tumor specific mutation detection in biofluid-derived EVs for diagnosis and monitoring [17].

Novel sequencing technologies have been used to identify tumor-specific molecular signatures in the EV-derived genetic cargo [18,19,20,21], but fusion detection from biofluids has not been previously reported. RNA-seq allows genome-wide transcriptome sequencing to identify novel gene fusions, and the latest advances have enhanced the sequencing capabilities to identify molecular alterations from ultra low-input RNA [22]. Here, we identify gene fusion transcripts in the extracellular vesicle RNA (EV RNA) derived from the plasma of patients with GBM. We used a QIAseq Targeted RNAscan Human Oncology Panel, which screens 232 known fusion-related genes, to identify RNA fusions. We explore the possibility of using RNA-seq-based fusion discovery to identify known and novel fusion transcripts in tumor tissue and the matched plasma of patients with GBM.

## 2. Results

### 2.1. Experimental Design and Overview of the Library Preparation Methodologies

**Figure 1:**
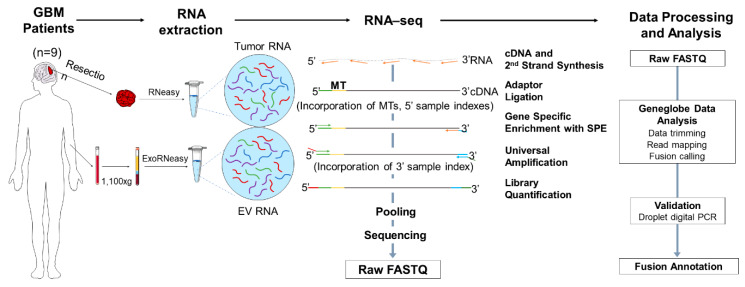
Overview of RNA-seq-based gene fusion discovery workflow. The RNA was extracted from the tumor tissue and matched plasma of patients with glioblastoma (GBM) (*n* = 9) and plasma of healthy controls (*n* = 10) and was sequenced using the QIAseq Targeted RNAscan Human Oncology Panel. Schematic illustration of the workflow depicting the RNA extraction, library preparation, sequencing, data processing, analysis and final validation using droplet digital PCR and fusion annotation. GBM, glioblastoma; EV RNA, extracellular vesicle RNA; MT, molecular tag; SPE, single primer extension.

A cohort of adult GBM patients with prior molecular characterization (*n* = 9) and healthy controls (*n* = 10) were included in the RNA-seq exploratory fusion discovery study. Our GBM patient population included *n* = 3 patients with gene fusions (P1–P3) and *n* = 6 (P4–P9) patients with no gene fusions, as reported by Massachusetts General Hospital (MGH) Solid Fusion Assay. The demographic, histologic and molecular characteristics of the GBM patient cohort and the demographics of healthy controls are summarized in Table 1**.** The gene fusions analyzed by the MGH Solid Fusion Assay and RNA-seq are listed in Table S1 and Table S2, respectively. The RNA-seq analysis of the GBM tumor tissue and matched plasma of patients and healthy controls was performed using a QIAseq Targeted RNAscan Human Oncology Panel (Figure 1). Firstly, the RNA was extracted from tumor tissue using an RNeasy Mini kit, with 30 minutes of on-column DNase digestion using the RNase-Free DNase kit to eliminate the genomic DNA (gDNA). The RNA was isolated from 2 mL of plasma using an ExoRNeasy Maxi kit. All the RNA samples were analyzed using an Agilent 2100 Bioanalyzer to assess the integrity of the RNA extracted from the tumor tissue and plasma (Figure S1, Table S3).

The typical workflow of the RNA-seq library preparation and computational analysis is illustrated in Figure 1. In summary, the RNA samples are initially converted to first strand cDNA, and a second strand synthesis is used to generate double stranded cDNA (ds-cDNA). This ds-cDNA is then end-repaired, dA-tailed and ligated to a sequencing platform-specific adapter containing unique molecular tags (MTs) and sample indexes. These MTs enable original targets to be recognized by a unique sequence barcode that allows for accurate identification and quantification. Subsequently, the barcoded adapter-ligated cDNA molecules are subjected to a limited target-barcode enrichment with a single primer extension (SPE) for target enrichment. This enrichment ensures that the intended targets are sufficiently enriched to be represented in the final library. A universal PCR amplification is then performed to further amplify the library and add a second sample index. The sample libraries were generated after the library preparation and analyzed using an Agilent 2100 Bioanalyzer. The average length of the sample libraries of the tumor tissue and plasma is 400–500 bp and 350–400 bp, respectively (Figure S2). The sample libraries were quantified and pooled at equimolar ratio for sequencing.

A sequencing data analysis was performed in the GeneGlobe Data Analysis Center (Figure 1), and mapping metrics are shown in Table S6. The number of mapped reads were comparable between the tumor tissue and plasma samples. The average RNA Control Primers MT counts in the plasma sample libraries were lower than the 300 MT-cutoff defined by GeneGlobe, indicating a low diversity of RNA specimens in the plasma samples. The percentage of overall mapped reads longer than 200 base pairs (mapped reads% > 200 bp) was significantly lower in the plasma sample libraries than in the tumor tissue libraries, which could affect the sensitivity of fusion calling in plasma samples. The fusions detected using RNA-seq were further validated using droplet digital (ddPCR)**.** A summary of the fusion calling and ddPCR validation results is shown in Table S4.

### 2.2. Detection of Fusions in Tumor Tissue and Matched Plasma of Patients with GBM and Plasma of Healthy Controls

**Figure 2:**
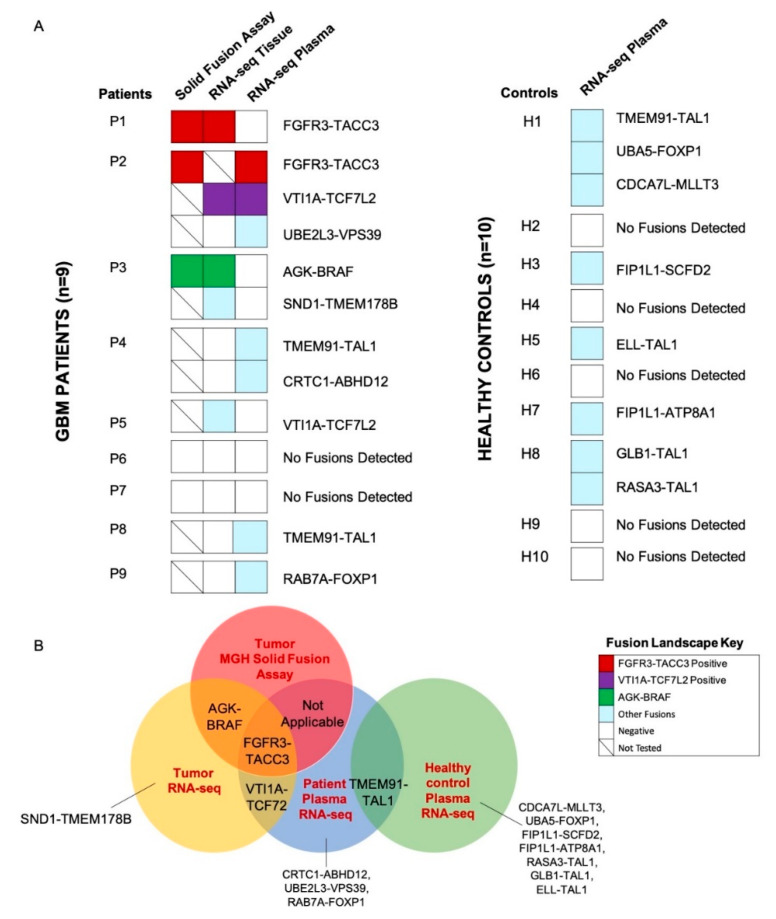
Tissue and plasma RNA fusion landscape. (**A**) Oncoprint depicting tumor tissue and plasma RNA fusions in the study cohort (GBM patients, *n* = 9, left; healthy controls, *n* = 10, right). (**B**) Summary of fusions detected by the Massachusetts General Hospital (MGH) Solid Fusion Assay of tumor tissue, RNA-seq of the tumor tissue and plasma of GBM patients and RNA-seq of the plasma of healthy controls.

We screened the GBM tumor tissue and matched plasma of *n* = 9 patients to identify the gene fusions identified by the MGH Solid Fusion Assay and additional novel fusion transcripts (Figure 2A). Patients P1 (FGFR3-TACC3), P2 (FGFR3-TACC3) and P3 (AGK-BRAF) had gene fusions reported by the MGH Solid Fusion Assay. The corresponding fusion transcripts were identified in the tumor tissue of patients P1 and P3. A tumor tissue RNA-seq analysis of P2 was not performed due to inadequate tumor tissue. Patients P4-P9 did not have gene fusions reported by MGH Solid Fusion Assay. Interestingly, the RNA-seq analysis of the tumor tissue revealed no fusion transcripts in patients P4-P9, except in patient P5 (VTI1A-TCF7L2). Novel fusion transcripts were identified in the tumor tissue of P2 (VTI1A-TCF7L2), P3 (SND1-TMEM178B) and P5(VTI1A-TCF7L2) (Figure 2B). Collectively, there was a 100% concordance between the common gene fusions screened by the MGH Solid Fusion Assay and the RNA-seq. In total, >1 fusion transcripts were detected in the tumor tissue of 22% (2/9) of GBM patients.

The RNA-seq analysis of the matched plasma samples of the GBM patients (P1-P3) with gene fusions reported in the MGH Solid Fusion Assay revealed fusions in P2 (FGFR3-TACC3, VTI1A-TCF7L2). No fusion transcripts were detected in P1 and P3. FGFR3-TACC3, VTI1A-TCF7L2 fusion transcripts were detected in both the tissue and matched plasma of patient P2 (Figure 2A). The analysis of the plasma samples of GBM patients (P4–P9) with no gene fusions reported in the MGH Solid Fusion Assay revealed fusion transcripts in P4 (TMEM91-TAL1, CRTC1-ABHD12), P8 (TMEM91-TAL1) and P9 (RAB7A-FOXP1). No fusions were identified in patients P5, P6 and P7. In total, >1 fusion transcripts were detected in the plasma of 22% (2/9) of GBM patients.

**Figure 3:**
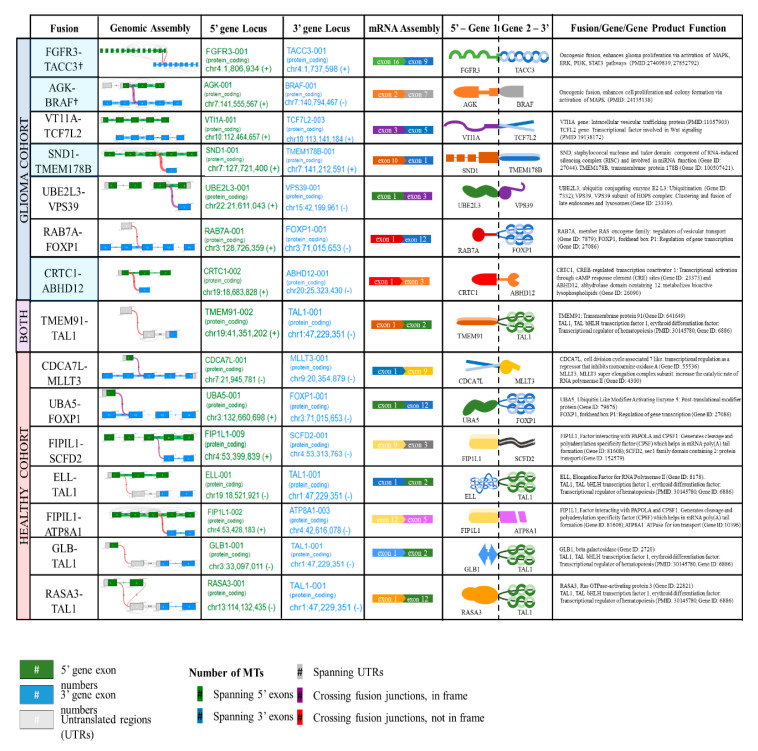
Annotation of gene fusions at genomic, transcriptomic and fusion protein level. Summary of genomic assembly, gene locus (QIAseq RNAscan Analysis Report generated on GeneGlobe), mRNA assembly, hypothetical fusion protein diagram and gene fusion/gene function. †, fusions with documented oncogenic function. Light blue shade indicates the GBM fusions in hotspot regions.

Eight novel fusion transcripts were detected by an RNA-seq analysis in the healthy controls H1 (TMEM91-TAL1, CDCA7L-MLLT3, UBA5-FOXP1), H3 (FIP1L1-SCFD2), H5 (ELL-TAL1), H7 (FIP1L1-ATP8A1) and H8 (GLB-TAL1, RASA3-TAL1). TMEM91-TAL1 was the only fusion transcript commonly reported in both the patient and healthy control plasma (Figure 2B). The supporting MTs and ddPCR validation copies of the sample libraries of tumor tissue and plasma samples are reported in Table S4. The hypothetical assembly of fusions at genomic, transcriptomic and proteomic levels is further annotated in Figure 3. Furthermore, 63% (5/8) of the fusion transcripts identified in GBM patients occur in chromosomes 4, 7 and 19, which have been previously described in literature as fusion hotspots (Figure 3). Additionally, 50% (4/8) of fusion transcripts identified in the healthy controls occurred in these regions. 

### 2.3. Longitudinal Follow-Up of Patient 2

**Figure 4:**
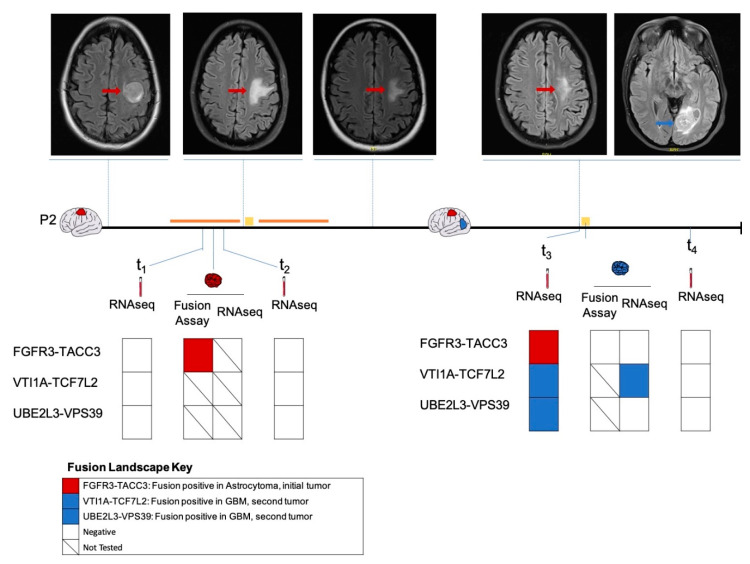
Longitudinal monitoring of fusion profile in patient P2. The fusion profiles of the initial frontal diffuse astrocytoma (indicated in red), plasma samples pre-surgery (t_1_) and post-surgery (t_2_) as well as the subsequent occipital GBM (indicated in blue), plasma samples pre-surgery (t_3_) and one month post-surgery (t_4_) as determined by MGH Solid Fusion Assay, RNA-seq were represented. Magnetic resonance images of the corresponding time points along the disease course were shown. Surgical procedures are indicated using a yellow square, administration of chemoradiation is indicated using an orange line.

Patient 2 presented with seizures secondary to a left frontal mass (Figure 4), with imaging features suggestive of a high-grade glioma. A biopsy was deferred in light of the patient’s comorbidities. The patient was empirically treated with chemoradiation for three months. However, due to the radiographic evidence of tumor progression, the patient underwent a subtotal resection to establish the diagnosis. A histopathologic analysis revealed an IDH wildtype diffuse astrocytoma positive for FGFR3-TACC3 fusion. The patient subsequently underwent the remaining cycles of chemoradiation. We analyzed the plasma collected pre-surgery (t_1_) and post-surgery (t_2_) and identified no fusions on RNA-seq analysis. There was no sufficient tumor tissue available from this time point for sequencing analysis.

Approximately 1.5 years following initial diagnosis, the patient developed a new left occipital lesion, unrelated to the previous tumor. MRI revealed an independent new left occipital mass in addition to the residual frontal diffuse astrocytoma. The patient underwent a subtotal resection with pathology revealing a new isocitrate dehydrogenase (IDH) wild-type GBM negative for FGFR3-TACC3 by the MGH Solid Fusion Assay (Figure 4). An RNA-seq analysis of the GBM tumor detected a VTI1A-TCF7L2 fusion. Plasma collected pre-surgery (t_3_) also revealed a VTI1A-TCF7L2 fusion. Additionally, fusions UBE2L3-VPS39 and FGFR3-TACC3 were also detected. A plasma RNA-seq one month later revealed no fusions. We hypothesized that the FGFR3-TACC3 fusion identified in the plasma at t_3_ was derived from the initial FGFR3-TACC3-positive diffuse astrocytoma. 

## 3. Discussion

RNA fusion transcripts are major drivers of cancer, and their accurate detection and monitoring is critical to advancing clinical care. Gene fusions occur in approximately 30–50% of patients with GBM [3], and understanding the characteristics of these fusions can be critical in developing personalized diagnostic and therapeutic modalities. Advances in sequencing technologies have opened up the possibility of screening a wide range of fusions, identifying novel oncogenic driver gene fusions or passenger fusions, which can be potential biomarkers [23,24,25]. Currently, tissue-based genetic tumor profiling is used to classify disease and guide therapy. The standard biopsy is associated with surgical complications and residual comorbidities, and therefore obtaining multiple biopsy sections routinely to monitor the disease over time and therapy is not always feasible. Liquid biopsy is an attractive alternative for the detection and monitoring of genetic alterations in biofluids, including blood, urine, cerebrospinal fluid (CSF), saliva etc., providing a minimally invasive platform by which to monitor brain tumors. Recent studies have shown the possibility of sequencing EV RNA in various biofluids to identify distinct molecular signatures [18,19,20,21], but the identification of gene fusions from plasma has not been reported.

Here we used a targeted RNA-seq panel (QIAseq Targeted RNAscan Human Oncology Panel) to screen the GBM tissue and matched plasma samples of nine GBM patients for both previously known and novel RNA fusions, demonstrating the feasibility of using an RNA-seq-based liquid biopsy to profile the transcriptome level gene fusions in EVs derived from the plasma of patients with GBM. There was a 100% concordance between the common gene fusions detected by MGH Solid Fusion Assay and RNA-seq, which was both reassuring and indicative of the specificity of the RNA-seq assay. We observed a clustering of the novel gene fusions VTI1A-TCF7L2 and SND1-TMEM178B along with the commonly reported FGFR3-TACC3 and AGK-BRAF fusions in the GBM tissue of patients P1-P3, who were fusion-positive by the MGH Solid Fusion Assay. Only one fusion, VTI1A-TCF7L2, was identified in the GBM tissue of patients P4–P9, who were fusion-negative by the MGH Solid Fusion Assay. This indicates the increased propensity of certain GBM tumors to harbor multiple fusions in their tissue due to genomic instability. Five novel fusion transcripts TMEM91-TAL1, CRTC1-ABHD12, VTI1A-TCF7L2, UBE2L3-VPS39 and RAB7A-FOXP1 were identified in the plasma of GBM patients. Eight novel fusion transcripts TMEM91-TAL1, CDCA7L-MLLT3, UBA5-FOXP1, FIP1L1-SCFD2, ELL-TAL1, FIP1L1-ATP8A1, GLB1-TAL1 and RASA3-TAL1 were identified in healthy plasma. Interestingly, TMEM91-TAL1 was the only fusion common in both plasma cohorts.

The detection of the FGFR3-TACC3 fusion transcript in the plasma is of important clinical value. This fusion is formed by the confluence of its fusion partners that code for fibroblast growth factor receptor 3 (FGFR3) and transforming acidic coiled-coil (TACC3). This chimeric protein localizes to mitotic spindles, resulting in chromosomal segregation defects and the induction of aneuploidy, leading to cancer [26]. This oncogenic fusion is known to occur in 1.2% to 8.3% of patients with GBM [3,27], and activates mitogen-activated protein kinase (MAPK), extracellular signal-regulated kinase (ERK), phosphatidylinositol-4,5-bisphosphate 3-kinase (PI3K) and the signal transducer and activator of transcription 3 (STAT3) pathways to promote tumor proliferation [26,28,29,30,31]. Drugs targeting FGFR kinase activity have shown promise in preclinical and clinical trials [9,26,32,33]. A real-time polymerase chain reaction (RT-PCR)-based screening assay has been developed to identify all the possible FGFR3-TACC3 variants in GBM tumor tissue, despite its structural heterogeneity at the genomic level and variability at the mRNA level [29]. Our limited capability of detection of this fusion transcript in plasma could be attributed to its heterogeneity at the genomic and transcriptomic levels, as well as the heterogeneity of plasma samples and EVs. Nevertheless, our preliminary results indicate the possibility of the detection of FGFR3-TACC3 in plasma, opening new avenues of liquid biopsy for the detection of FGFR3-TACC3 fusion transcripts to aid in the diagnosis and monitoring of FGFR3-TACC3-positive GBMs. Additionally, this fusion is reported in the context of other systemic cancers, including bladder, head and neck, lung, nasopharyngeal, esophageal squamous cell and cervical cancers [26]. Thus, an FGFR3-TACC3 liquid biopsy assay would have far-reaching benefits.

The VTI1A-TCF7L2 fusion transcript was identified in the GBM tissue of patients P2 and P3; additionally, this fusion was also identified in the plasma of patient P2 in a longitudinal setting. Although the exact function of the fusion protein has not been described, VTI1A-TCF7L2 has been implicated in several cancers, including lung, colorectal cancer (CRC), breast cancer and glioma [34,35]. The VTI1A (vesicle transport through interaction with t-SNAREs 1) gene encodes a soluble N-ethylmaleimide-sensitive fusion protein-attachment protein receptor that functions in intracellular trafficking [36], while the TCF7L2 (transcription factor 7-like 2) gene product is a high mobility group box-containing transcription factor that plays a key role in the Wnt-signaling pathway [37]. The TCF7L2 gene is frequently mutated in CRC [37] and has been demonstrated to enhance cell proliferation [38]. Considering the role of the VTI1A gene in intracellular vesicular trafficking, this fusion is an attractive candidate for EV-based fusion biomarking. Further functional studies are required to explore the oncogenic mechanisms and clinical relevance of this recurrent fusion in glioma and other cancers.

Shah et al. analyzed the transcriptome of 185 GBM tumors and identified two major genomic hotspots at the chromosomal locations 7p and 12q. Additional regions, including chromosomes 1, 4, 6 and 19, also revealed a high frequency of fusions. Furthermore, they showed that nearly 20% of GBMs have a propensity to harbor more than one fusion, and the majority of the detected fusions were private fusions occurring only in one patient [3]. We detected >1 fusion transcripts in the tumor tissue and plasma of 22% (2/9) of GBM patients and in 20% (2/10) of the healthy controls. Interestingly, 63% (5/8) of the fusion transcripts detected in GBM patients occurred in these hotspots, specifically in chromosomes 4, 7 and 19. These chromosomal locations are likely unstable in GBM, with high a propensity of forming deletion bridges which connect these amplified regions, generating multiple fusion transcripts.

We also detected four novel fusions involving the gene domain TAL1, including TMEM91-TAL1, ELL-TAL1, GLB1-TAL1 and RASA3-TAL1. Interestingly, all of them were detected in the plasma of healthy controls, while TMEM91-TAL1 was detected in the plasma of two patients with GBM and a healthy control. The TAL1 gene is an essential transcriptional regulator of hematopoiesis, in particular for the specification of hematopoietic lineages [39]. TAL1 is also implicated as a key transcriptional factor involved in the development of T-cell acute lymphoblastic leukemia [40]. Similarly, the MLLT3 (mixed lineage leukemia, translocated to 3, also known as AF9) gene is a critical regulator of hematopoietic stem cell self-renewal, and also implicated in leukemias [41,42,43]. The detection of fusions involving the TAL1 and MLL3 fusion domains in the plasma of healthy controls could be a consequence of their wide roles in hematopoiesis. As none of the healthy controls have a history of leukemia, it is highly likely that these TAL1 fusions are passenger fusions. The detection of passenger somatic mutations in the cell-free DNA or white blood cells of healthy individuals is not uncommon [44,45,46,47], and it could be indicative of an evolutionary path to increase protein diversity [48] or tumor formation [46,49]. This strengthens the argument that cancer progression is a fine balance of driver and passenger mutations [50]. The relevance of the fusion transcripts identified in the healthy controls is largely unknown, and further studies are needed to decipher their functional significance in health and disease. Finally, the low frequency of hematopoietic fusion detection in GBM patients compared to healthy controls could implicate the abundance of tumor-specific EVs in the plasma of GBM patients, thereby hindering any passenger fusion detection [51,52,53]. Additionally, the justification of using normal human plasma as a negative control cohort, in the light of evidence that fusion transcripts also occur in healthy tissue, is subject to question. The assumption that healthy controls to have no fusion transcripts could deviate and undermine our confidence in the true fusions detected in GBM patients [4].

In addition, we report several novel gene combinations (H1: CDCA7L-MLLT3, P3: SND1-TMEM178B), of which individual fusion partners have been previously reported in the context of several cancers, including MLLT3 in leukemia [54], SND1 (staphylococcal nuclease and tudor domain) in lung cancer [55,56]. These are probably coincidental and lack clinical relevance. Further investigation might provide insights into the relevance and the function of these fusions, if any.

Patient 2 initially had a frontal diffuse astrocytoma positive for an FGFR3-TACC3 fusion, and underwent chemoradiation and a subtotal resection. Later, the patient developed an occipital GBM, which had no fusions as reported by a MGH Solid Fusion Assay. RNA-seq of the occipital GBM tumor tissue revealed the presence of a VTI1A-TCF7L2 fusion. A plasma analysis prior to the resection also revealed a VTI1A-TCF7L2 fusion in addition to an FGFR3-TACC3 fusion. The FGFR3-TACC3 fusion likely originated from the initial residual frontal diffuse astrocytoma. Although the FGFR3-TACC3 fusion was not detected at the time points t_1_, t_2_ or t_4_, this could probably be due to the heterogeneity in the plasma EV RNA and low sensitivity of the RNA-seq platform for low-input RNA sequencing. Nevertheless, our results indicate the feasibility of RNA-level fusion detection in plasma. This offers new avenues to expand liquid biopsy-based methods for fusion transcript detection in tumors and matched plasma for diagnostic and monitoring purposes.

One limitation of this study includes the small fragment length of EV RNA (<200 nt), which might undermine the sensitivity of the assay, as larger fragments are more favorable for fusion detection. Moreover, the low RNA yield and heterogeneity of the RNA isolated from plasma EVs limits fusion discovery and requires further optimization of the fusion discovery platforms to identify genetic alterations from the low-input EV RNA. This heterogeneity of the EV RNA between biological replicates (multiple plasma samples from the same patient) challenges the reproducibility in fusion discovery. However, this is a common limitation for any EV-based biomarker discovery study [57]. Furthermore, the fusions reported may be influenced by the biofluid in question. For instance, a multitude of fusions identified in plasma could be traced to hematopoiesis, which might mask GBM -specific fusion detection. Our analysis uses a targeted panel which is limited in the range of the fusion transcripts screened. In addition, adopting a GeneGlobe Data Analysis platform with default settings as a tool for the data analysis in this study also limits the flexibility of the data processing and fusion calling. Furthermore, the RNA-seq analysis cannot provide insights into the fusion-generating mechanism [4]. Finally, our study is limited by the small sample size. A large statistically powered study with well-defined cohorts, identifying whole transcriptome RNA fusions and the corresponding whole genome DNA fusions would be required to verify, validate and better depict the plasma GBM fusion landscape in order to better distinguish true fusions from sequencing and bioinformatics artifacts and identify clinically relevant recurrent fusions.

This proof-of-principle study demonstrates the feasibility of utilizing targeted RNA-seq for the fusion transcript profiling of EV RNA derived from the plasma of patients with GBM. The detection of fusion transcripts in biofluids can open avenues to aid in the management of patients with GBM in the contexts of diagnosis, prognosis, monitoring as well as patient stratification for novel clinical trials. Further studies with more sensitive sequencing technologies are required to explore the clinical implications of fusion transcript detection in plasma.

## 4. Materials and Methods 

### 4.1. Study Subject Characteristics

The study population (*n* = 9) consisted of pathology-confirmed IDH wild-type GBM (WHO grade IV) patients ranging from 41 to 74 years who underwent surgery at Massachusetts General Hospital (MGH). Tumor tissue and matched plasma samples were collected. The patient demographics, pathological diagnosis and molecular characteristics are reported in Table 1. Plasma samples from healthy family members (*n* = 3) as well as unrelated healthy volunteers (*n* = 7) were also collected. The demographics of the healthy controls are reported in Table 1. All the samples were collected with informed consent under appropriate protocols approved by Partners institutional review board (Protocol# 2017P001581).

### 4.2. Tumor Tissue Processing and RNA Extraction 

The tumor tissue was microdissected and suspended in RNAlater (Ambion, Austin, TX, USA) and stored at −80 ℃ before use. The frozen tumor tissue was thawed at room temperature and the RNA was extracted using an RNeasy Mini kit according to the manufacturer’s instructions, with 30 min of on-column DNase digestion using the RNase-Free DNase kit (both Qiagen, Hilden, Germany) to eliminate the genomic DNA (gDNA). Each tumor RNA sample was eluted in 40 μL nuclease-free water (Invitrogen, Carlsbad, CA, USA) and stored at −80 ℃ before use.

### 4.3. Plasma processing 

Whole blood samples were collected using BD Vacutainer® blood collection tubes (BD Life Science, Franklin Lakes, NJ, USA). Within 2 h of collection, the samples were centrifuged at 1100× *g* for 10 min and filtered using 0.8 μm filters prior to being aliquoted into cryovials (Thermo Fisher Scientific, Waltham, MA, USA). All the aliquoted samples were stored at −80 °C.

### 4.4. Plasma EV RNA Extraction 

The RNA was isolated from 2 mL of plasma from the patients as well as healthy controls using the ExoRNeasy Maxi kit (Qiagen) according to the manufacturer’s instructions. The EV RNA was eluted in 14 μL of nuclease-free water and stored at −80 ℃ before use.

### 4.5. RNA Quantification and Quality Control 

The concentration of the tumor RNA samples was determined using the Qubit RNA HS Assay Kit on a Qubit 4 fluorometer (both Thermo Fisher Scientific). The RNA was assessed for quality and integrity using the profiles generated by an Agilent Bioanalyzer. For the tumor tissue, the RNA was diluted to meet the recommended range and assessed using an Agilent RNA Nano Kit; the plasma RNA was directly assessed using an Agilent RNA Pico Kit according to the manufacturer’s instructions (both Agilent Technologies, Santa Clara, CA, USA).

The tumor RNA was also assessed for gDNA contamination by a GAPDH RT-qPCR (cutoff Ct 30) using a 100 ng tumor RNA template, 2× Power SYBR™ Green PCR Master Mix (Applied Biosystems, Foster, KY, USA) and 4µM of forward and reverse primers (Invitrogen, 5’-GAAGGTGAAGGTCGGAGTC-3’ and 5’-GAAGATGGTGATGGGATTTC-3’) in a total reaction mix of 25 µL. The thermocycling conditions were as follows: 95 °C for 10 min, 40 cycles of 95 °C for 15 s and 60 °C for 1 min with data collection. The cycling was performed on a StepOnePlus Real-Time PCR System (Applied Biosystems). The Ct values were analyzed using the StepOne Software (v2.3) with auto settings.

### 4.6. Library Preparation and Sequencing 

A QIAseq Targeted RNAscan Human Oncology Panel and QIAseq 12-Index (both Qiagen) were used for the library prep and sample indexing. An amount of 100 ng of the tumor RNA and 5 μL of the EV RNA were used as an input for the library preparation, as per the manufacturer’s instructions. The sample libraries were diluted to meet the recommended concentration range and assessed using a DNA High Sensitivity Chip (Agilent Technologies) on an Agilent Bioanalyzer to determine the size distribution, and were quantified on a Qubit 4 fluorometer using a Qubit dsDNA HS Assay kit (Thermo Fisher Scientific). The molarity of the sample libraries was calculated based on the concentration and median library size. The quantified libraries were normalized to 4 nM and pooled at equimolar ratios. The sequencing libraries were finally denatured and diluted to 6 pM according to the guidelines (Illumina, San Diego, CA, USA). Paired end sequencing was performed on a MiSeq Sequencer to generate FASTQ files only, using a MiSeq Reagent Kit V2, 300 cycles (Illumina), with 231 cycles for read 1 and 71 cycles for read 2, as per the panel instructions. 

### 4.7. Sequencing Data Processing 

An integrated Qiagen panel-specific data analysis pipeline was adopted here for data processing (Figure 1). The raw individual FASTQ files were uploaded to GeneGlobe Data Analysis Center using a NGS Analysis Tool designed for the QIAseq Targeted RNAscan Panel with default settings (Qiagen, Hilden, Germany). The MT sequences were first extracted from the R2 reads for quantification and trimmed off along with adapters and other stable sequences using cutadapt software (version 1.9.1). Reads were mapped to the reference genome GENECODE Release 26 (*Homo sapiens*, GRCh38.p10) using STAR software (version 2.5.2a). The R1 reads containing gene-specific primers were first mapped with downstream endogenous bases; the unmapped segments were mapped in the 2nd pass. Quality metrics, including mapping status, RNA control primers, mapped read length and splice distance statistics were generated for a data evaluation. 

### 4.8. Fusion Calling and Reportable Range 

The GeneGlobe data analysis pipeline integrates a list of predefined filters to avoid reporting artifacts caused by sequence homology, read-though RNA or signal noise. For a fusion to be called by the GeneGlobe data analysis pipeline, the fusions must (1) contain exons from different genes within one read or read-pair; (2) be at least 12 base pairs long; (3) pass all filters or be flagged with at most one filter; (4) be curated fusions, which are extensively described in the literature (and will be reported even with limited evidence). Our final reporting strategy includes (1) curated fusions and (2) high confidence fusions, which passed all filters defined by GeneGlobe. Low confidence fusions, which have not passed all the GeneGlobe defined filters or are not curated are not reported as true fusions. Fusions COL1A1-COL1A2 and HACL1-COLQ are reported by GeneGlobe as a result of the bioinformatics artifact and are thus excluded from our analysis [58]. The summary of fusion calling results and the number of MTs supporting the fusion are provided in Table S4. Supporting reads for the fusion were used to generate contigs using SPAdes software (version 3.10.1) to obtain a fusion consensus sequence.

### 4.9. Validation of Fusions by Droplet Digital PCR 

We validated all the positive fusions reported by RNA-seq using a droplet digital PCR (ddPCR) assay. The primers (Invitrogen) were designed to target the fusion sequence using Primer-Blast (NCBI) and were shown in Table S5 Supplementary Materials, Dataset. S2. ddPCR amplification was performed in duplicate, using 1 µL of a 4 nM sequencing libraries template, 10 µL 2× QX200 ddPCR EvaGreen Supermix (Bio-Rad, Hercules, USA) and 1 µL of each 2.5 nM primer (Invitrogen) in a total reaction mix of 20 µL. The QX200 AutoDG Automatic Droplet generator (both Bio-Rad) was used to generate droplets. The thermocycling conditions were as follows: 95 °C for 5 min, 40 cycles of 95 °C for 30 s and 60 °C for 1 min, followed by 4 °C for 5 min and 90 °C for 5 min and held at 4 °C until further processing. The droplets were counted and analyzed using the QX200 droplet reader, and a QuantaSoft analysis (Bio-Rad) was performed to acquire absolute quantification data. The average fusion copies per/μL were taken and the gates were set based on the fusion wild-type samples in the 1-D amplification plot (Figure S3). The summary of the ddPCR copies/μL supporting the validation of fusions is provided in Table S4.

## 5. Conclusions

In this study, we used targeted RNA-seq to screen tumor tissue and matched plasma samples from GBM patients for the presence of known and novel fusion transcripts. We successfully detected a range of previously reported oncogenic fusions (FGFR3-TACC3, AGK-BRAF) as well as novel fusions, including VTI1A-TCF7L2 and SND1-TMEM178B. The fusion transcripts FGFR3-TACC3 and VTI1A-TCF7L2 were detected in both the tissue and matched plasma. Furthermore, we identified an FGFR3-TACC3 fusion transcript in the patient plasma in a longitudinal setting. Our study demonstrates the feasibility of RNA-seq-based fusion transcript detection and paves a path for liquid biopsy-based fusion transcript profiling for diagnosis and longitudinal monitoring.

## Figures and Tables

**Table 1:** Demographics of Glioblastoma Multiforme (GBM) patients and healthy control cohorts.

IDs	Age	Sex	Diagnosis	IDH	MGMT	EGFR	MET	TERT	CDKN2A
**P1**	72	F	GBM	WT	Methylated	Unamplified	Unamplified	Mutant	Loss
**P2**	41	F	GBM	WT	Methylated	NK	NK	WT	Maintained
**P3**	52	F	GBM	WT	Methylated	NK	NK	Mutant	Maintained
**P4**	73	F	GBM	WT	Unmethylated	Unamplified	Unamplified	Mutant	Loss
**P5**	71	M	GBM	WT	Unmethylated	Amplified	Amplified	WT	Maintained
**P6**	61	F	GBM	WT	Unmethylated	Unamplified	Unamplified	WT	Maintained
**P7**	68	F	GBM	WT	Unmethylated	Amplified	NK	Mutant	Maintained
**P8**	65	F	GBM	WT	Methylated	Unamplified	Unamplified	WT	Loss
**P9**	74	F	GBM	WT	Methylated	Unamplified	Amplified	WT	Maintained
**H1**	NK	NK	-	-	-	-	-	-	-
**H2**	24	F	-	-	-	-	-	-	-
**H3**	NK	NK	-	-	-	-	-	-	-
**H4**	NK	NK	-	-	-	-	-	-	-
**H5**	25	M	-	-	-	-	-	-	-
**H6**	24	F	-	-	-	-	-	-	-
**H7**	24	F	-	-	-	-	-	-	-

Abbreviations: GBM, glioblastoma; WT, wildtype; IDH, isocitrate dehydrogenase; EGFR, epidermal growth factor receptor; MET, MET proto-oncogene; TERT, telomerase reverse transcriptase; CDKN2A, Cyclin Dependent Kinase Inhibitor 2A; NK, not known.

## Supplementary Materials

The following are available online at https://www.mdpi.com/2072-6694/12/5/1219/s1. Figure S1: Representative RNA Bioanalyzer profiles; Figure S2: Representative library Bioanalyzer profiles’ Figure S3: Representative ddPCR 1-D amplification plot; Table S1: Genes screened by MGH Solid Fusion Assay; Table S2: Genes screened by QIAseq Targeted RNAscan Panel; Table S3: tumor RNA and EV RNA profiles; Table S4: Summary of fusion calling results; Table S5: Primers design for fusion transcript droplet digital PCR validation; Table S6: Overview of GeneGlobe RNA-seq mapping metrics.
