# Supplementary material for: Novel Gene Fusions in Glioblastoma Tumor Tissue and Matched Patient Plasma
Source: Cancers (Basel). 2020 May 13;12(5):1219. doi: 10.3390/cancers12051219 (PMC7281415; doi:10.3390/cancers12051219)
Supplement: Supplementary file 1 [file cancers-12-01219-s001.pdf]

cancers

MDPI

Novel Gene Fusions in Glioblastoma Tumor Tissue and Matched Patient Plasma

Lan Wang [1,†], Anudeep Yekula [1,†], Koushik Muralidharan [1], Julia L. Small [1], Zachary S. Rosh [1], Keiko M. Kang [1,2], Bob S. Carter [1,*] and Leonora Balaj [1,*]

[1] Department of Neurosurgery, Massachusetts General Hospital and Harvard Medical School, Boston, MA 02115, USA. lanwang.amber@gmail.com (L.W.); ayekula@mgh.harvard.edu (A.Y.); kmuralidharan@mgh.harvard.edu (K.M.); jsmall6@mgh.harvard.edu (J.S.); Zrosh@mgh.harvard.edu (Z.R.); kmkang@mgh.harvard.edu (K.M.K.)

[2] School of Medicine, University of California San Diego, San Diego, CA 92092, USA

† These authors contributed equally.

* Correspondence: bcarter@mgh.harvard.edu (B.S.C.); Balaj.Leonora@mgh.harvard.edu (L.B.)

Supplementary

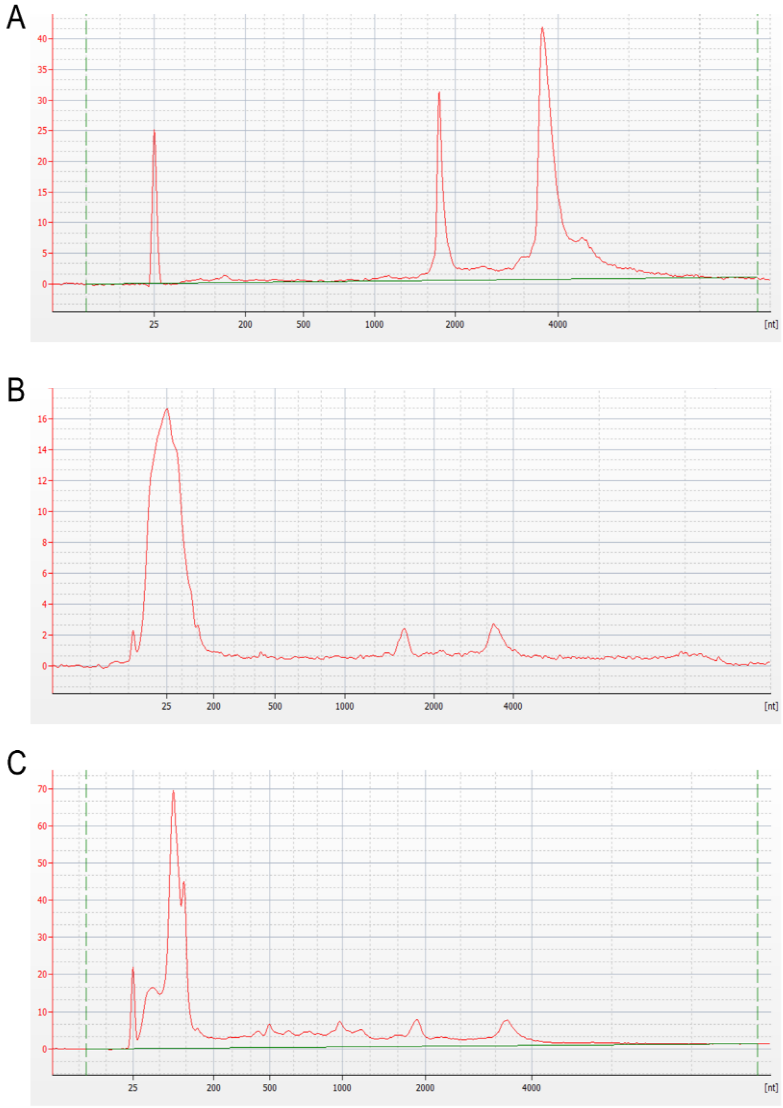

Figure S1. Representative Bioanalyzer profiles of RNA extracted from (A) GBM tumor, (B) patient plasma and (C) healthy control plasma.

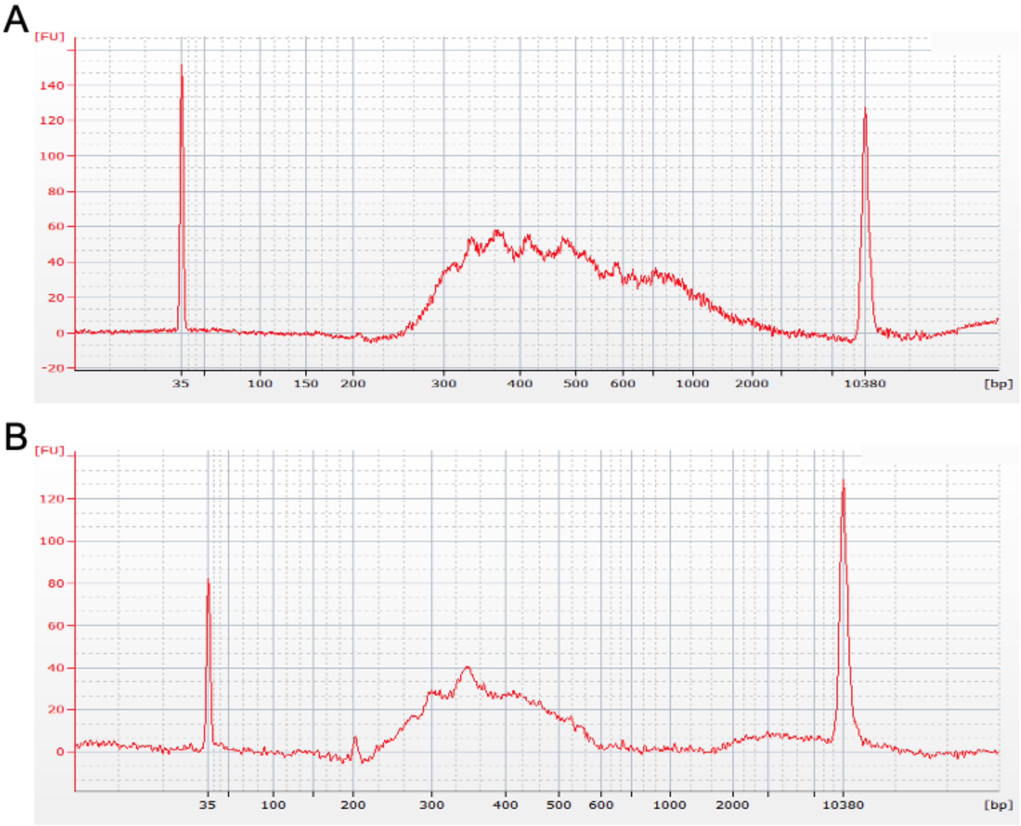

Figure S2. Representative DNA Bioanalyzer profiles of libraries extracted from (A) GBM tumor, (B) patient plasma.

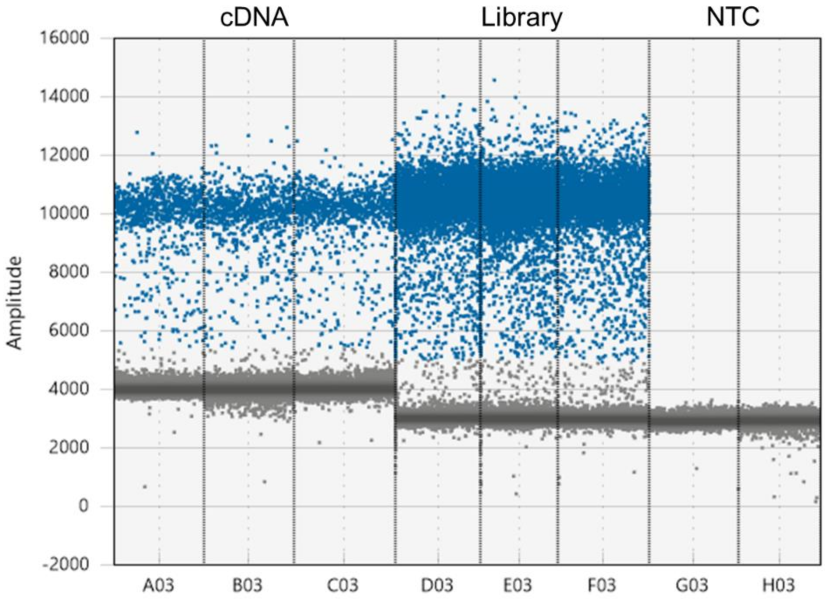

Figure 3. 1-D amplification plot of AGK-BRAF fusion droplet digital PCR validation assay of GBM tumor cDNA (left), Sample library (center) from patient P3 and No template control (right).

Table 1. MGH Solid Fusion Assay screens for fusions involving the above genes.

AKT3, ALK, AR, ARHGAP26, AXL, BRAF, BRD3, BRD4, CSF1, CSF1R, EGFR, ERG, ESR1, ESRRA ETV1, ETV4, ETV5, ETV6, EWSR1, FGFR1, FGFR2, FGFR3, FGFR3, FGR, INSR, JAZF1, MAML2, MAST1, MAST2, MET, MET, MSMB, MUSK, MYB, NOTCH1, NOTCH2, NRG1, NTRK1, NTRK2, NTRK3 NUMBL, NUTM1, PDGFRA, PDGFRB, PIK3CA, PKN1, PPARG, PRKCA, PRKCB, RAF1, RELA, RET, ROS1, RSPO2, RSPO3, TERT, TFE3, TFEB, THADA, TMPRSS2

Table 2. QIAseq Targeted RNAscan Panel screens for fusions involving the above genes.

ABI1, ABL1, ABL2, ACTN4, AFF1, AFF3, AGK, AKAP9, ALK, ARHGAP26, ARHGEF12, ASPSCR1, ATF1, ATIC, AUTS2, BAG4, BAIAP2L1, BCOR, BCR, BIRC, BRAF, CARS, CASC5, CBFA2T3, CBFB, CBL, CCDC6, CCDC88C, CD74, CEP170B, CEP89, CHIC2, CIAO1, CLCN6, CLTC, CNTRL, COL1A1, COPA, CREB1, CREBBP, CRTC1, CUX1, DAZL, DCTN1, DDIT3, DDX10, DEK, DHH, ELL, ELN, EML1, EML4, EP300, EPS15, ERC1, ERG, ESRP1, ETV1, ETV4, ETV6, EWSR1, EZR, FAM131B, FCHSD1, FGFR1, FGFR1OP, FGFR2, FGFR3, FIP1L1, FLI1, FLT3, FN1, FNBP1, FOXO1, FOXO4, FOXP1, FRYL, FRYL, FUS, GAS7, GATA1, GATM, GLIS2, GNAI1, GOLGA5, GOPC, GOT1, GPHN, HACL1, HERPUD1, HIP1, HLF, HOOK3, HOXA9, HOXD13, JAK2, KAT6A, KDM5A, KIF5B, KLC1, KMT2A, KRAS, LMNA, LNP1, LNP1, LRIG3, LSM14A, MALT1, MAML2, MECOM, MKL1, MKRN1, MLF 1.00, MLLT1, MLLT1, MLLT10, MLLT11, MLLT3, MLLT6, MLLT6, MN1, MNX1, MPRIP, MRPS14, MSN, MYB, MYH11, MYO1F, MYO5A, NCOA2, NCOA4, NCOA4, NDE1, NPM1, NRG1, NSD1, NTRK1, NTRK3, NUMA1, NUP214, NUP98, P2RY8, PAPSS1, PAX3, PAX5, PAX7, PAX8, PBX1, PCM1, PDGFB, PDGFRA, PDGFRB, PHF23, PICALM, PLAG1, PML, PPARG, PPFIBP1, PRCC, PRDM16, PRKAR1A, PRKG2, PRRX1, PSIP1, PTPRR, PWWP2A, RAF1, RANBP2, RAP1GDS1, RARA, RBM15, RCSD1, RET, RHEBL1, RNF130, ROS1, RPL22, RPN1, RUNX1, RUNX1T1, SDC4, SEC31A, SEPT2, SEPT5, SEPT6, SEPT9, SET, SFPQ, SFPQ, SLC34A2, SLC45A3, SND1, SQSTM1, SRGAP3, SS18, SSX1, SSX2, SSX2B, SSX2B, STAT5B, STIL, STRN, TACC1, TACC3, TAF15, TAF15, TAL1, TCF3, TCF7L2, TET1, TFE3, TFG, TMPRSS2, TOP 1.00, TPM3, TPM4, TPR, TRIM24, TRIM27, TRIM33, TRIP11, UBE2L3, UBE3C, USP42, VCL, VTI1A, WHSC1L1, WT1, ZBTB16, ZMIZ1, ZMYM2, ZNF384, ZNF703, ZSCAN30

Table 3. RNA profiles indicated by Agilent Bioanalyzer 2100 Expert Software and/or Qubit 4 Fluorometer. Tumor tissue RNA concentration were determined by Qubit 4 Fluorometer. RIN, RNA fragments % > 200 bp of all RNA samples and plasma RNA concentration were calculated by 2100 Expert Software with baseline correction. RIN, RNA Integrity Number.

Sample cohort	Patient/Healthy control ID	RIN	Concentration (ng/µL)	RNA fragment% >200bp
**Tumor tissue**	P1	8.0	45.4	92%
	P2	9.9	538.0	96%
	P3	7.7	53.9	80%
	P4	9.3	424.0	98%
	P5	9.1	209.0	88%
	P6	9.0	392.7	88%
	P7	8.7	70.4	94%
	P8	8.9	138.4	95%
	P9	9.6	81.2	96%
**Patient matched plasma**	P1	2.0	0.074	10%
	P2(t_1)	2.6	1.820	14%
	P2(t_2)	2.7	0.981	10%
	P2(t_3)	4.8	0.153	14%
	P2(t_4)	3.9	0.370	19%
	P3	6.8	0.062	6%
	P4	4.1	0.120	14%
	P5	1.4	0.082	21%
	P6	2.0	0.041	5%
	P7	5.5	0.181	17%

	P8	5.3	0.135	24%
	P9	4.2	0.202	17%
**Healthy control plasma**	H1	2.9	1.206	11%
	H2	2.5	2.112	12%
	H3	2.6	1.869	13%
	H4	2.6	1.499	14%
	H5	2.7	4.274	14%
	H6	3.1	3.369	17%
	H7	2.8	3.187	16%
	H8	3.1	3.685	17%
	H9	2.3	2.610	14%
	H10	3.0	5.598	19%

Table S4. Summary of fusion calling and droplet digital PCR (ddPCR) validation results. Fusions identified by MGH Solid Fusion Assay and RNA-seq are listed including the fusion categories (curated, high confidence or low confidence fusions) and number of supporting molecular tags (MTs). The corresponding ddPCR validation results for the curated and high confidence fusions. (NA, Not Available).

Sample Cohort	Patient/ Healthy Control ID	MGH Solid Fusion Assay Fusions	RNA-seq Fusions	Fusion Category	Supporting MTs	ddPCR Positive Droplet Concentration (copies/μL)
**GBM Tumor Tissue**	P1	FGFR3-TACC3	FGFR3-TACC3	Curated	751	65550
			RANBP2-RGPD8	Low confidence	150	-
			PICALM-SYTL2	Low confidence	12	-
			COL1A1-COL1A2	Low confidence	11	-
	P2	None	VTI1A-TCF7L2	Curated	1	151.5
			COL1A1-COL1A2	Low confidence	136	-
			COL1A1-COL2A1	Low confidence	27	-
	P3	AGK-BRAF	AGK-BRAF	Curated	625	51650
			SND1-TMEM178B	High confidence	425	29250
			CLU-TOP1	High confidence	7	-
			PTPRZ1-ELN	High confidence	8	NA
			RANBP2-RGPD8	Low confidence	269	-
			EWSR1-FUS	Low confidence	10	-
			PICALM-SYTL2	Low confidence	7	FAIL
	P4	None	RANBP2-RGPD8	Low confidence	212	-
			MAP1B-TOP1	Low confidence	6	-
			CD74-CLU	Low confidence	5	-
			CLU-TOP1	Low confidence	5	-
			EWSR1-FUS	Low confidence	6	-
	P5	None	VTI1A-TCF7L2	Curated	1	128.5
	P6	None	COL1A1-COL1A2	Low confidence	17	-
	P7	None	None	NA	0	-
	P8	None	FGFR3-TACC3	Curated	2	-
			RANBP2-RGPD8	Low confidence	171	-
			SYN2-PPARG	Low confidence	4	-
			ATRNL1-MLLT10	Low confidence	3	-
	P9	None	RANBP2-RGPD8	Low confidence	248	-
**Patient Matched Plasma**	P1	Not tested	None	NA	0	-
	P2 (t_1)	Not tested	None	NA	0	-
	P2 (t_2)	Not tested	None	NA	0	-
	P2 (t_3)	Not tested	FGFR3-TACC3	Curated	2	507600
			VTI1A-TCF7L2	Curated	2	16615
			UBE2L3-VPS39	High confidence	55	18500
	P2 (t_4)	Not tested	None	NA	0	-
	P3	Not tested	CD74-GID8	Filtered	57	-
	P4	Not tested	TMEM91-TAL1	High confidence	12	33600
			CRTC1-ABHD12	High confidence	49	8800
	P5	Not tested	None	NA	0	-
	P6	Not tested	None	NA	0	-
	P7	Not tested	RUNX1-RUNX3	Low confidence	4	-

	P8	Not tested	TMEM91-TAL1	High confidence	7	7725
			FIP1L1-SCFD2	Low confidence	6	-
	P9	Not tested	RAB7A-FOXP1	High confidence	8	8065
			RANBP2-RGPD8	Filtered	13	-
			NPM1-FBXO38	Filtered	4	-
**Healthy Control Plasma**	H1	Not tested	TMEM91-TAL1	High confidence	22	49400
			CDCA7L-MLLT3	High confidence	12	50400
			UBA5-FOXP1	High confidence	14	17350
			FIP1L1-SCFD2	Low confidence	6	-
	H2	Not tested	None	NA	0	-
	H3	Not tested	FIP1L1-SCFD2	High confidence	8	34850
	H4	Not tested	ACTN1-ACTN4	Low confidence	8	-
	H5	Not tested	ELL-TAL1	High confidence	4	NA
	H6	Not tested	None	NA	0	-
	H7	Not tested	FIP1L1-ATP8A1	High confidence	64	NA
	H8	Not tested	GLB1-TAL1	High confidence	6	NA
			RASA3-TAL1	High confidence	6	NA
	H9	Not tested	None	NA	0	-
	H10	Not tested	None	NA	0	-

Table S5. Primers design for fusion transcript droplet digital PCR validation. F and R primers for fusion transcript sequences used for droplet digital PCR target amplification. F: forward primers; R: reverse primers.

Fusion name		Breakpoint Location	Primers	Sequences
**TMEM91-TAL1**	5'	TMEM 91 exon 1, chr19:41,351,202 (+)	F	CGTGCGAGACGAGACACAT
	3'	TAL1 exon 2, chr1:47,229,351 (-)	R	CTGAGAGGCCTGCAGTTACG
**CRTC1-ABHD12**	5'	CRTC1 exon 1, chr19:18,683,828 (+)	F	ACAATCAGAAGCAGGCGGAG
	3'	ABHD12 exon 3, chr20:25,323,430 (-)	R	TGATTCAAACCTTGATCCTGTGG
**FIP1L1-SCFD2**	5'	FIP 1L1 exon 12, chr4:53,428,183 (+)	F	GCACTGCTCCACCTCTGATT
	3'	SCFD2 exon 5, chr4:53,145,582 (-)	R	GCTGCTTTAACACAACGGACA
**UBE2L3-VPS39**	5'	UBE2L3 exon 3, chr22:21,611,043 (+)	F	CCAAACATCGACGAAAAGGGG
	3'	VPS39 exon 2, chr15:42,199,961 (-)	R	TGGTTCCCACAAGAAGCCATT
**RAB7A-FOXP1**	5'	RAB7A exon 1, chr3:128,726,359 (+)	F	GTTTAGTCTCCTCCTCGGCG
	3'	ROXP1 exon 12, chr3:71,015,653 (-)	R	TGGCCACTTGCATACACCAT
**CD74-GID8**	5'	CD74 exon 9, chr5:150,402,152 (-)	F	CAGTCCCCATGTGAGAGCAG
	3'	GID8 exon 5, chr20:62,944,739 (+)	R	TCCGTGGTAGACCGAATCCT
**CDCA7L-MLLT3**	5'	CDCA7L exon 1, chr7:21,945,781 (-)	F	GCCCGGTTAGGAAGAATGGA
	3'	MLLT3 exon 9, chr9:20,354,879 (-)	R	TGCTTATCTGATTTGCTTTGCTT
**UBA5-FOXP1**	5'	UBA5 exon 1, chr3:132,660,698 (+)	F	GAGCTCAGAGGTGGTGGATTC
	3'	FOXP1 exon 12, chr3:71,015,653 (-)	R	CAGCCTGGCCACTTGCATA
**FIP1L1-SCFD2**	5'	FIP1L1 exon 9, chr4:53,399,839 (+)	F	TTGTTCAAGACTGGGCTTCCA
	3'	SCFD2 exon 3, chr4:53,313,763 (-)	R	GCAGGTTTTCTCTGCTTGCC
**VTI1A--TCF7L2**	5'	VTI1A exon 3, chr10:112,464,657 (+)	F	TCCGAGAGATACCACCCCAA
	3'	TCF7L2 exon 5, chr10:113,141,184 (+)	R	GCACCACTGGCACTTTGTTAG
**FGFR3-TACC3**	5'	FGFR3 exon 16, chr4:1,806,934 (+)	F	GACCTGGACCGTGTCCTTACC
	3'	TACC3 exon 9, chr4:1,737,598 (+)	R	TGGAGCAGGTCCACTATAGGTC
**AGK-BRAF**	5'	AGK exon 2, chr7:141,555,567 (+)	F	ACGCTTCGAAATCACTGGAAG
	3'	BRAF exon 7, chr7:140,794,467 (-)	R	TGAAGGAGACGGACTGGTGA
**SND1-TMEM178B**	5'	SND1 exon 10, chr7:127,721,400 (+)	F	ACGATTCACCTGTCCAGCAT
	3'	TMEM178B exon 1, chr7:141,212,591 (+)	R	TAGGGCATGCCACTCATCCT
**PTPRZ1-ELN**	5'	PTRPZ1 exon 21, chr7:122,040,979 (+)	F	GCCTGACATGGGAGTACCAG
	3'	ELN exon 3, chr7:74,037,707 (+)	R	GGCTTAAGAGGTTTGCCTCCA
**PICALM-SYTL2**	5'	PICALM exon 19, chr11:85,974,708 (-)	F	AAACCCCTTTGGCCCTGTATC
	3'	SYTL2 exon 1, chr11:85,758,114 (-)	R	CAGCACACTCACTCTCTGGT
**ELL-TAL1**	5'	ELL exon 1, chr19:18,521,921 (-)	F	GCGCTGAAGGAGGATAGGAG
	3'	TAL1 exon 2, chr1:47,229,351 (-)	R	CTGAGAGGCCTGCAGTTACG
**FIP1L1-ATP8A1**	5'	FIP1L1 exon 12, chr4:53,428,183 (+)	F	GCACTGCTCCACCTCTGATT
	3'	ATP8A1 exon 5, chr4:42,616,078 (-)	R	GCGTTTGTTTCTTGTTCACTGC
**GLB1-TAL1**	5'	GLB1 exon 1, chr3:33,097,011 (-)	F	CGGGGTTCCTGGTTCGCAT
	3'	TAL1 exon 2, chr1:47,229,351 (-)	R	TTACGCTGCGGTGTGGTCC
**RASA3-TAL1**	5'	RASA exon 1, chr13:114,132,435 (-)	F	GTCTTCCAGAGCGTGAAGATCA
	3'	TAL1 exon 2, chr1:47,229,351 (-)	R	CTGAGAGGCCTGCAGTTACG

Table S6. Overview of RNA-seq mapping metrics generated by GeneGlobe Data Analysis Center. RNA Control Primers refer to a set of RNA control primers targeting housekeeping genes that can be used as an indicator of RNA diversity (QIAseq™ Targeted RNAscan Data Analysis Handbook, Qiagen).

Sample Cohort	Patient/ Healthy Control ID	Raw Reads/ Sample	Mapped Reads/ Sample	Average RNA Control Primers	Reads% > 200 bp
GBM Tumor Tissue	P1	3,254,559	3,084,254	4,712.00	31.04%
	P2	3,251,846	3,177,744	4,291.80	33.15%
	P3	3,739,661	3,488,775	2,857.80	23.65%
	P4	2,854,488	2,729,137	5,061.00	36.34%
	P5	2,120,682	2,025,337	4,000.80	35.87%
	P6	2,891,115	2,795,359	3,230.20	33.38%
	P7	2,563,322	2,432,636	5,364.20	35.42%
	P8	2,982,206	2,825,331	6,289.80	32.92%
	P9	3,109,352	2,970,338	6,059.50	43.20%
Matched Patient Plasma	P1	2,394,700	2,250,364	82.5	17.40%
	P2 (t1)	2,741,135	2,600,817	116.2	20.31%
	P2 (t2)	2,404,068	2,277,630	129.2	18.60%
	P2 (t3)	3,227,719	3,060,547	184.8	9.90%
	P2 (t4)	3,227,967	3,044,622	157.5	21.12%
	P3	2,742,402	2,536,385	174.5	21.15%
	P4	2,345,261	2,231,584	152.2	18.83%
	P5	2,745,203	2,608,936	153.5	21.52%
	P6	1,910,362	1,813,593	136.5	19.80%
	P7	1,429,178	1,352,839	115.5	19.30%
	P8	2,694,475	2,546,389	163.2	18.60%
	P9	3,157,529	3,005,320	202.2	20.50%
Healthy Control Plasma	H1	6,352,456	5,887,078	195.5	20.08%
	H2	5,955,852	5,607,881	290.8	19.90%
	H3	4,668,412	4,385,014	227.5	19.50%
	H4	5,681,289	5,211,746	232.8	17.67%
	H5	2,519,716	2,377,286	233	17.70%
	H6	2,016,223	1,906,711	127.2	18.30%
	H7	2,753,480	2,614,837	183.8	19.84%
	H8	2,809,201	2,663,076	255.8	20.53%
	H9	2,543,422	2,420,411	182.2	20.12%
	H10	2,617,655	2,487,716	180.2	19.80%
